# *In-silico* evaluation of Malawi essential medicines and reactive metabolites for potential drug-induced toxicities

**DOI:** 10.1186/s40360-021-00499-6

**Published:** 2021-06-16

**Authors:** Ibrahim Chikowe, Alfred Chipanda Phiri, Kirios Patrick Mbewe, Dunstan Matekenya

**Affiliations:** 1 Pharmacy Department, College of Medicine, University of Malawi, Blantyre, Malawi; 2 The World Bank Group, DECDG, Washington, DC USA

**Keywords:** Screening, Drug-induced toxicity, Computer-aided

## Abstract

**Background:**

Drug-induced toxicity is one of the problems that have negatively impacted on the well-being of populations throughout the world, including Malawi. It results in unnecessary hospitalizations, retarding the development of the country. This study assessed the Malawi Essential Medicines List (MEML) for structural alerts and reactive metabolites with the potential for drug-induced toxicities.

**Methods:**

This *in-silico* screening study used StopTox, ToxAlerts and LD-50 values toxicity models to assess the MEML drugs. A total of 296 drugs qualified for the analysis (those that had defined chemical structures) and were screened in each software programme. Each model had its own toxicity endpoints and the models were compared for consensus of their results.

**Results:**

In the StopTox model, 86% of the drugs had potential to cause at least one toxicity including 55% that had the potential of causing eye irritation and corrosion. In ToxAlerts, 90% of the drugs had the potential of causing at least one toxicity and 72% were found to be potentially reactive, unstable and toxic. In LD-50, 70% of the drugs were potentially toxic.

Model consensus evaluation results showed that the highest consensus was observed between ToxAlerts and StopTox (80%). The overall consensus amongst the three models was 57% and statistically significant (*p* < 0.05).

**Conclusions:**

A large number of drugs had the potential to cause various systemic toxicities. But the results need to be interpreted cautiously since the clinical translation of QSAR-based predictions depends on many factors. In addition, inconsistencies have been reported between screening results amongst different models.

**Supplementary Information:**

The online version contains supplementary material available at 10.1186/s40360-021-00499-6.

## Background

Structural alerts, also known as toxicophores, are highly reactive molecular fragments that can cause adverse effects either directly or after going through metabolism or biotransformation by human enzymes and gut microbiota [1, 2]. Reactive metabolites are molecules with high chemical reactivity that are formed via biotransformation by human enzymes or gut microbiota. Structural alerts and reactive metabolites have the potential of causing drug-induced toxicities [3].

The concept of structural alerts has been used widely in the pharmaceutical industry, and regulatory bodies like the US Food and Drug Administration (FDA), as well as in drug discovery circles, because they are easy to understand and they are also inexpensive to apply. In the regulatory process, these tests are requested by regulatory authorities for all new chemical compounds before taking them to human clinical trials. However, structural alerts data are prone to bias, and more robust approaches, such as Quantitative Structure-Activity Relationships (QSAR) modeling and/or Chemical Biological Read-Across (CBRA) models are recommended to improve the accuracy of toxicity prediction [4]. Interest in structural alerts studies has also increased with their inclusion in the Registration, Evaluation, Authorization, and Restriction of Chemicals (REACH) regulation or law by the European Council and European Parliament in 2006, which made structural alerts an essential part of chemical safety assessment and regulation. In this law, computational approaches are recommended within integrated testing strategies for hazard prediction and safe design of new chemicals [3–5]. In the drug discovery area, all the lead compounds with structural alerts and potential to produce reactive metabolites are taken to have toxicity risk and are therefore removed from the list or their structures are modified to get rid of the structural alerts [6–8]. However, this may bring challenges if the structural alerts are part of the pharmacophore (the functional group responsible for drug candidate activity) as it is for structural alerts such as furan, thiophene, nitroaromatic, phenol, and aniline that are also known to be pharmacophores [9]. They can give rise to a pharmacological activity or provide pharmacokinetic benefits.

Several studies have been conducted in countries like the USA to evaluate the databases of their commonly-used drugs for structural alerts and to relate the IADR predictions to clinical data to find out which drugs fit the models and which do not [10, 11]. In some cases, this may be used to warn health care providers to be cautious when prescribing these medicines and watch out for any indicated toxicity in their patients. Clinical feedback may reveal whether the models are correct or not, and this may provide room for improvement in the models or declaration of the safety of the molecules. Such studies are, however, lacking in Malawi. Therefore, this study evaluated the Malawi Essential Medicines List for structural alerts and reactive metabolites with the potential for drug-induced toxicities.

## Methods

This was an *in-silico* study that used virtual screening of the drugs on the Malawi Essential Medicines List (MEML) for the structural alerts that have the potential to cause drug-induced toxicity. A database of drugs used in Malawi was created from all the medicines listed in the MEML. This involved the collection of drug names, chemical structures, and simplified molecular-input line-entry systems (SMILES). This generated 296 drugs. Many software programmes were identified and these included DEREK, TOPKAT [12], ToxAlerts [13], Bioalerts [14], Toxtree [4] and StopTox [15]. StopTox was selected as the main software due to it being recent and easy to use as well as for its implementation of QSAR models developed using the best practices for validation and development required by Organisation for Economic Cooperation and Development (OECD). In addition, StopTox employs a wide range of endpoints using the largest publicly available and well-curated animal data. Lastly, StopTox provides the prediction of fragment contribution, which shows maps of fragments that are predicted to increase or decrease the toxicity profile. However, other toxicity screening models, namely ToxAlerts and LD-50 values were used for comparison with the StopTox results.

**Fig. 1:**
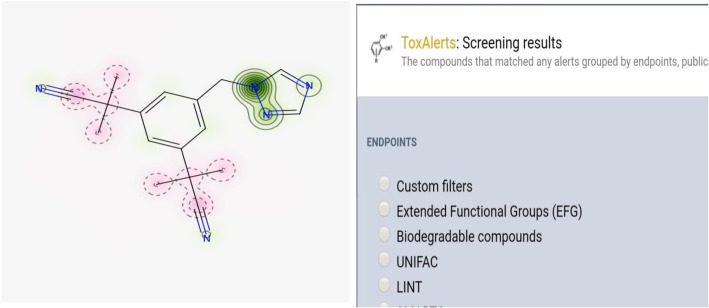
Map of predicted fragment contribution and structural alerts screening of Anastrazole using STopTox and ToxAlerts respectively

Chemical structures were represented in an Excel file as simplified molecular-input line-entry system (SMILES) strings with identifiers for the compounds. MarvinSketch software available at https://marvinjs-demo.chemaxon.com/latest/demo.html was used to draw the structures and insert them in the excel sheet where SMILES strings were also copied. Structures, LD-50 values and SMILES for the drugs in the MEML [16] were obtained from Drug Bank [17] and other online sources. The SMILES for each drug were then fed into the StopTox and ToxAlerts softwares that was freely available on https://stoptox.mml.unc.edu/ [18] and https://ochem.eu//alerts/screen.do?render-mode=full respectively. The LD-50 values were searched from both the Drug Bank and a large dataset of acute oral toxicity data created for testing *in-silico* models by the U.S Environmental Protection Agency’s (EPA) National Center for Computational Toxicology that was accessed through its CompTox Chemicals Dashboard that was freely available on https://comptox.epa.gov/dashboard . If no data were found for certain drugs in the database, information was collected from chemical data sheets available online. Different LD-50 values were found for each drug that depended on the routes of administration and the animal used. In addition, there was no drug that had LD-50 values for all the routes of administration for all the animal models; that made it difficult to choose a specific LD-50 value to use for comparison. So a range of LD-50 values was compiled. Interpretation of values was based on the EPA’s 4-category hazard classification and the largest values in the range were used for interpretation since they mostly corresponded to acute oral LD-50 values [19–21]. Figure 1 below shows the structure of Anastrazole after its SMILE was run in the StopTox and ToxAlerts softwares.

The drugs were categorized into different groups of toxicities they caused. The data included acute inhalation toxicity, acute oral toxicity, acute dermal toxicity, eye irritation and corrosion, skin sensitization, skin irritation, and corrosion and consensus (overall toxicity). The number and percentages of the drugs with toxicities for each system, and overall, were derived using Microsoft Excel.

Consensus of the three toxicity predicting models was also evaluated using descriptive statistics parameters as well as specialized consensus measuring parameters. In descriptive statistics, the number of agreements between every two models as well as amongst all the three models were counted and converted to percentage. In the consensus evaluation, confusion matrix and binomial test were used. In confusion matrix, StopTox was taken as a true value while the other models (ToxAlerts and LD-50 values) were taken as predicted values. For the test between ToxAlerts and LD-50 values, the former was used as a true value while the latter was used as a predicted value. In binomial test, the number of trials was defined as N representing the number of drugs tested. Success was scenarios where all the three models agreed with probability *p*, while failure was cases where the models did not agree. X was considered as the probability of the models agreeing being binomial test to check whether the observed proportions of successful events deviated significantly from chance. Then a one sided binomial test was run with a hypothesized probability of success at *a* = 0.05. The confusion matrix and binomial tests were done using scipy: a Python package.

## Results

The Malawi Essential Medicines List contains at least 330 chemical substances that are used as drugs, or sanitation and hygiene chemical substances. Of the 330 drugs, 34 substances such as Fresh Frozen Plasma did not have SMILES and could not be fed into the software. The remaining 296 drugs had SMILES and were fed into the StopTox and ToxAlerts software to be screened. Table 1 shows the systemic toxicities for the drugs screened. Supplementary Table 1 shows the drugs and their respective toxicities using the three toxicity predicting models.

**Table 1:** Systemic toxicities of the MEML drugs and their proportions

Variable	Frequency (n)	Percentage (%)	
Acute inhalation toxicity	54	18	Acetic acid, Amethocaine, Amitriptyline, Anastrozole, Benzhexol, Biperiden, Brimonidine, Bupivacaine, Carbex/Selegiline, Carbimazole, Cetrimide/Cetrimonium, Chlorhexidine, Chlorambucil, Chloroquine, Diazepam, Chlorpheniramine maleate, Chlorpromazine, Cimetidine, Clofazimine, Clomiphene citrate, Clotrimazole, Cyclizine, Edrophonium chloride, Emedastine, Ethambutol, Nitrate, Ethionamide, Ethosuximide, Feruxomide, Gentian violet, Halofantrine, Hydrogen peroxide, Lidocaine, Lindane, Lumefantrine, Metformin, Nifedipine, Orphenadrine, Oxymetazoline, Pethidine, Pilocarpine, Pralidoxime, mesylate, Prochlorperazine, Procyclidine, Proguanil, Promethazine, Pyrimethamine, Ropivacaine, Silver nitrate, Sulphur/Octasulfur, Suxamethonium, Tamoxifen, Tropicamide, Zinc oxide.
Oral acute toxicity	143	48	Acetylsalicylic acid (Aspirin), Acetic acid, Benzhexol, Adriamycin, Adrenaline/Epinephrine, Alcuronium chloride, Allopurinol, Amethocaine, Amitriptyline, Amlodipine, Amodiaquine, Anastrozole, Atovaquone, Atropine, Azathioprine, Baclofen, Barium sulphate, Benzyl benzoate, Betaxolol, Biperiden, Bismuth chelate/Bismuth subsalicylate, Brimonidine, Brinzolamide, Busulphan, Carbamazepine, Carbex/Selegiline, Carbimazole, Cetirizine, Chlorhexidine, Chlorambucil, Chlordiazepoxide, Chloroquine, Chlorpheniramine maleate, Chlorpromazine, Clarithromycin, Clotrimazole, Codeine phosphate, Cyclizine, Cyclopentolate, Dapsone, Cyclophosphamide, Diazepam, Diclofenac sodium, Digoxin, Dihydrocodeine tartrate, Dinoprostone, Dorzolamide, Econazole, Edrophonium chloride, Efavirenz, Emedastine, Ergometrine maleate, Ethionamide, Ethosuximide, Ethyl chloride, Ferrous sulphate + folic acid, Feruxomide, Fluocinolone acetonide Fluorometholone, Fluoxetine, Fluphenazine, Gentian violet, Glyceryl trinitrate, Haloperidol, Hydralazine HCL, Hydrogen peroxide, Hyoscine, Indomethacin, butylbromide/Butylscopolamine, Ifosphamide, Iodine, Isoniazid, Ketoconazole, Ketotifen, Lidocaine, Lindane, Loperamide, Lumefantrine, Magnesium sulphate, Mebendazole, Mefenamic acid, Mefloquine, Melarsprol, Melphalan, Metformin, Methotrexate, Metoclopramide, Miconazole, Misoprostol, Morphine sulphate, Mycophenolate mofetil, Nalidixic acid, Naloxone HCL, Neostigmine methylsulphate, Nevirapine, Niclosamide, Nifedipine, Nitrate, Nitrofurantoin, Ofloxacin, Orphenadrine, Oxymetazoline, Paracetamol, Paraldehyde, Pethidine, Phenobarbital, Phenothiazine, Phenylephrine, Phenoxymethylpenicillin, Pilocarpine, Podophyllin resin, Potassium Chloride, Potassium permanganate, Pralidoxime mesylate, Praziquantel, Prazosin, Procarbazine, Prochlorperazine, Procyclidine, Proguanil, Promethazine, Propranolol HCL, Pyrimethamine, Quinine, Reserpine, Risperidone, Salbutamol, Silver nitrate, Sodium Bicarbonate, Sodium chloride, Sulbutamol sulphate, Sulphur/Octasulfur, Tamoxifen, Tetracycline, Theophylline, Timolol, Tramadol, Travatan (Travoprost), Tropicamide, Vecuronium bromide, Verapamil, Vincristine, Warfarin sodium and Zinc oxide.
Acute dermal toxicity	58	20	Adrenaline/Epinephrine, Amethocaine, Amitriptyline, Baclofen, Bismuth chelate/Bismuth subsalicylate, Brimonidine, Carbex/Selegiline, Carbimazole, Cetrimide/Cetrimonium, Chlorhexidine, Chloroquine, Chlorpheniramine maleate, Chlorpromazine, Cimetidine, Clofazimine, Clomiphene citrate, Clotrimazole, Cyclizine, cyclophosphamide, Dithranol, Efavirenz, Ethionamide, Ethyl chloride, Fluocinolone acetonide, Fluorescein sodium, Gentian violet, Glyceryl trinitrate, Griseofulvin, Hydralazine HCL, Ibuprofen, Ifosphamide, Iodine, Isosorbide dinitrate, Ivermectin, Lidocaine, Lindane, Lumefantrine, Magnesium trisilicate, Metformin, Neostigmine methylsulphate, Nitrate, Orphenadrine, Pethidine, Phenothiazine, Phenylephrine, Podophyllin resin, Potassium Chloride, Potassium permanganate, Prochlorperazine, Proguanil, Silver nitrate, Sodium chloride, Sodium cromoglycate/Cromoglicic acid, Sulphur/Octasulfur, Suxamethonium, Tamoxifen, Warfarin sodium, Zinc oxide.
Eye irritation and corrosion	163	55	Acetazolamide, 5 Fluorouracil, Acetic acid, Acyclovir, Adriamycin, Albendazole, Allopurinol, Amethocaine, Amikacin, Aminophylline, Amlodipine, Amodiaquine, Amoxicillin, Amphotericin B, Ampicillin, Anastrozole, Atorvastatin, Azathioprine, Azithromycin, Barium sulphate, Bendrofluazide, Benzathine, Didanosine penicillin/Benzylpenicillin, Benzoic acid, Benzoyl peroxide, Bicalutamide, Bimatoprost, Bismuth chelate/Bismuth subsalicylate, Bismuth subgallate, Bleomycin, Brimonidine, Brinzolamide, Bromocriptine, Busulphan, Capreomycin, Captopril, Carbimazole, Cefepime, Cefotaxime, Ceftazidime, Ceftriaxone, Cetrimide/Cetrimonium, Chloramphenicol, Dorzolamide, Chlordiazepoxide, Cimetidine, Ciprofloxacin, Econazole, Clarithromycin, Clavulanic acid, Clindamycin, Clofazimine, Clotrimazole, Cloxacillin, Desferrioxamine, Diazepam, cyclophosphamide, Cycloserine, cyclosporine, Dithranol, Doxycycline, Enalapril, Ergometrine maleate, Ethosuximide, Ethyl chloride, Ferrous sulphate + folic acid, Feruxomide, Flucloxacillin, Fluconazole, Fluorescein sodium, Fluphenazine, Folic acid, Furosemide, Ganciclovir, Gastrografin/Diatrizoate, Gentamicin, Glibenclamide, Gliclazide, Glyceryl trinitrate, Hydrochlorothiazide, Hydroxocobalamin, Hyoscine, butylbromide/Butylscopolamine, Idoxuridine, Ifosphamide, Imipenem, Indomethacin, Iodine, Isosorbide dinitrate, Kanamycin, Ketoconazole, Lamivudine, Leucovorin, Lidocaine, Lumefantrine, Lumigan, (Bimatroprost), Magnesium sulphate, Mebendazole, Meropenem, Methotrexate, Metoclopramide, Metolazone, Metronidazole, Miconazole, Moxifloxacin, Mycophenolate mofetil, N-acetylcysteine, Nalidixic acid, Natamycin, Nedocromil sodium, Neostigmine methylsulphate, Nifedipine, Nimodipine, Nitrate, Nitrofurantoin, Nystatin, Ofloxacin, Omeprazole, Oxymetazoline, Paclitaxel, Paraldehyde, Phenobarbital, Phenoxymethylpenicillin, Phenytoin, Pilocarpine, Piperacillin, Potassium Chloride, Potassium permanganate, Pralidoxime mesylate, Praziquantel, Prazosin, Proguanil, Prohance/Gadoteridol, Promethazine, Pyrimethamine, Ranitidine, Reserpine, Rifampicin, Risperidone, Silver nitrate, Silver sulphadiazine, Sodium amidotrizoate/Diatrizoate, Sodium Bicarbonate, Sodium chloride, Sodium lactate/Lactic acid, Stavudine, Streptomycin, Sulfamethoxazole, Sulphadoxine, Sulphur/Octasulfur, Suramin, Taxotere/Docetaxel, Tazobactam, Tenofovir, Tetracycline, Theophylline, Thiamine, Timolol, Vancomycin, Vecuronium bromide, Vincristine, Vitamin B12, Zidovudine, Zinc oxide.
Skin sensitization	76	26	Adrenaline/Epinephrine, Amethocaine, Amitriptyline, Amodiaquine, Ampicillin, Anastrozole, Azithromycin, Baclofen, Barium sulphate, Benzathine, penicillin/Benzylpenicillin, Benzoyl peroxide, Benzyl benzoate, Betaxolol, Bismuth chelate/Bismuth subsalicylate, Busulphan, Captopril, Carbex/Selegiline, Carbidopa, Cetrimide/Cetrimonium, Chlorhexidine, Chloramphenicol, Chlordiazepoxide, Chloroquine, Chlorpromazine, Clofazimine, Clotrimazole, Cloxacillin, Cyclizine, Desferrioxamine, Diazepam, Dinoprostone, Dithranol, Edrophonium chloride, Ferrous sulphate + folic acid, Flucloxacillin. Fluconazole, Fluorescein sodium, Fluoxetine, Gentian violet, Glyceryl trinitrate, Halofantrine, Hydralazine HCL, Isosorbide dinitrate, Lumefantrine, Magnesium sulphate, Magnesium trisilicate, Methyldopa, Metronidazole, Miconazole, Mycophenolate mofetil, Naloxone HCL, Niclosamide, Nitrate, Nitrofurantoin, Orphenadrine, Oxymetazoline, Phenoxymethylpenicillin, Phenylephrine, Pilocarpine, Potassium permanganate, Pralidoxime mesylate, Prochlorperazine, Proguanil, Promethazine, Propranolol HCL, Pyridoxine, Pyrimethamine, Quinine, Ranitidine, Salbutamol, Silver nitrate, Silver sulphadiazine, Sulbutamol sulphate, Travatan (Travoprost), Vitamin K, Xalatan (Latanoprost).
Skin irritation and corrosion	39	13	Acetic acid, Amitriptyline, Artemether, Barium sulphate, Busulphan, Carbex/Selegiline, Cetrimide/Cetrimonium, Chlorpheniramine maleate, Chlorpromazine, Clotrimazole, cyclophosphamide, Ethyl chloride, Ferrous sulphate + folic acid, Feruxomide, Fluphenazine, Foscarnet, Hydrogen peroxide, Ifosphamide, Iodine, Lindane, Magnesium sulphate, Magnesium trisilicate, Nitrate, Orphenadrine, Paraldehyde, Phenothiazine, Potassium Chloride, Potassium permanganate, Prochlorperazine, Pyrimethamine, Silver nitrate, Sodium Bicarbonate, Sodium chloride, Sodium lactate/Lactic acid, Sodium thiosulphate/Thiosulphuric acid, Sodium Valproate, Sulphur/Octasulfur, Vitamin A, Zinc oxide.
drugs without indicated toxicities	42	14	Artesunate, Ascorbic acid, Atenolol, Beclomethasone dipropionate, Betamethasone, Bisacodyl, Calcium gluconate, Clobetasol propionate, Colchicine, Dexamethasone, Erythromycin, Estradiol/Estrogen, Ethinylestradiol, Fludrocortisone, Gabapentine, Glycerine/Glycerol, Hydrocortisone acetate, Hydroxyurea, Lactulose, Levodopa, Levonorgestrel, Levothyroxine, Lorazepam, Magnevist/Gadopentetic acid, Mannitol, Medroxyprogesterone acetate, Menthol, Methylprednisolone, Nicotinamide, Norethisterone, Norgestrel, Postinor-2/Levonorgestrel, Prednisolone, Progesterone, Pyrazinamide, Simvastatin, Sodium ascorbate, Spironolactone, Thyroxine sodium, Tranexamic acid, Triamcinolone acetonide, Trimethoprim.
Drugs with 1 toxicity only	117	40	Acetazolamide, Aspirin, 5 Fluorouracil, Acyclovir, Albendazole, Alcuronium chloride, Amikacin, Aminophylline, Amoxicillin, Amphotericin B, Artemether, Atorvastatin, Atovaquone, Atropine, Bendrofluazide, Benzoic acid, Bicalutamide, Bimatoprost, Bismuth subgallate, Bleomycin, Bromocriptine, Bupivacaine, Capreomycin, Carbamazepine, Carbidopa, Cefepime, Cefotaxime, Ceftazidime, Ceftriaxone, Cetirizine, Ciprofloxacin, Clavulanic acid, Clindamycin, Codeine phosphate, Cyclopentolate, Cycloserine, cyclosporine, Dapsone, Diclofenac sodium, Didanosine, Digoxin, Dihydrocodeine tartrate, Doxycycline, Enalapril, Ethambutol, Fluorometholone, Folic acid, Foscarnet, Furosemide, Ganciclovir, Gastrografin/Diatrizoate. Gentamicin, Glibenclamide, Gliclazide, Glyceryl trinitrate, Griseofulvin, Haloperidol, Hydrochlorothiazide, Hydroxocobalamin, Ibuprofen, Idoxuridine, Imipenem, Isoniazid, Ivermectin, Kanamycin, Ketotifen, Lamivudine, Leucovorin, Loperamide, Lumigan (Bimatoprost), Mefenamic acid, Mefloquine, Melarsprol melphalan, Meropenem, Methyldopa, Metolazone, Misoprostol, Morphine sulphate, Moxifloxacin, N-acetylcysteine, Natamycin, Nedocromil sodium, Nevirapine, Nimodipine, Nystatin, Omeprazole, Paclitaxel, Paracetamol, Phenytoin, Piperacillin, Procarbazine, Prohance/Gadoteridol, Pyridoxine, Rifampicin, Ropivacaine, Sodium amidotrizoate/Diatrizoate, Sodium cromoglycate/Cromoglicic acid, Sodium thiosulphate/Thiosulphuric acid, Sodium Valproate, Stavudine, Streptomycin, Sulfamethoxazole, Sulphadoxine, Suramin, Taxotere/Docetaxel, Tazobactam, Tenofovir, Thiamine, Tramadol, Vancomycin, Verapamil, Vitamin A, Vitamin B12, Vitamin K, Xalatan (Latanoprost), Zidovudine.
Drugs with 2 toxicities	65	22	Adriamycin, Allopurinol, Amlodipine, Ampicillin, Azathioprine, Azithromycin, Benzathine penicillin/Benzylpenicillin, Benzhexol, Benzoyl peroxide, Benzyl benzoate, Betaxolol, Biperiden, Brinzolamide, Captopril, Chlorambucil, Chloramphenicol, Clarithromycin, Clomiphene citrate, Cloxacillin, Desferrioxamine, Dinoprostone, Dorzolamide, Econazole, Efavirenz, Emedastine, Ergometrine maleate, Flucloxacillin, Fluconazole, Fluocinolone acetonide, Fluoxetine, Halofantrine, Hyoscine butylbromide/Butylscopolamine, Indomethacin, Ketoconazole, Mebendazole, Methotrexate, Metoclopramide, Metronidazole, Nalidixic acid, Naloxone HCL, Niclosamide, Ofloxacin, Phenobarbital, Podophyllin resin, Praziquantel, Prazosin, Procyclidine, Propranolol HCL, Quinine, Ranitidine, Reserpine, Risperidone, Salbutamol, Silver sulphadiazine, Sodium lactate/Lactic acid, Sulbutamol sulphate, Suxamethonium, Tetracycline, Theophylline, Timolol, Travatan (Travoprost), Vecuronium bromide, Vincristine, Warfarin sodium.
Drugs with 3 toxicities	28	9	Adrenaline/Epinephrine, Amodiaquine, Baclofen, Chlordiazepoxide, Cimetidine, Dithranol, Edrophonium chloride, Ethionamide, Ethosuximide, Fluorescein sodium, Fluphenazine, Hydralazine HCL, Hydrogen peroxide, Isosorbide dinitrate, Magnesium trisilicate, Metformin, Miconazole, Mycophenolate mofetil, Neostigmine methylsulphate, Nifedipine, Nitrofurantoin, Paraldehyde, Pethidine, Phenothiazine, Phenoxymethylpenicillin, Phenylephrinel, Sodium Bicarbonate, Tamoxifen.
Drugs with 4 toxicities	29	10	Acetic acid, Anastrozole, Barium sulphate, Bismuth chelate/Bismuth subsalicylate, Brimonidine, Busulphan, Carbimazole, Chlorhexidine, Chloroquine, Chlorpheniramine maleate, Clofazimine, Cyclizine, cyclophosphamide, Diazepam, Ethyl chloride, Ferrous sulphate + folic acid, Feruxomide, Gentian violet, Ifosphamide, Iodine, Lidocaine, Lindane, Magnesium sulphate, Oxymetazoline, Pilocarpine, Potassium Chloride, Pralidoxime mesylate, Promethazine, Sodium chloride.
Drugs with 5 toxicities	13	4	Amethocaine, Amitriptyline, Carbex/Selegiline, Cetrimide/Cetrimonium, Chlorpromazine, Lumefantrine, Orphenadrine, Potassium permanganate, Prochlorperazine, Proguanil, Pyrimethamine, Sulphur/Octasulfur, Zinc oxide.
Drugs with 6 toxicities	3	1	Clotrimazole, Nitrate, Silver nitrate.

**Fig. 2:**
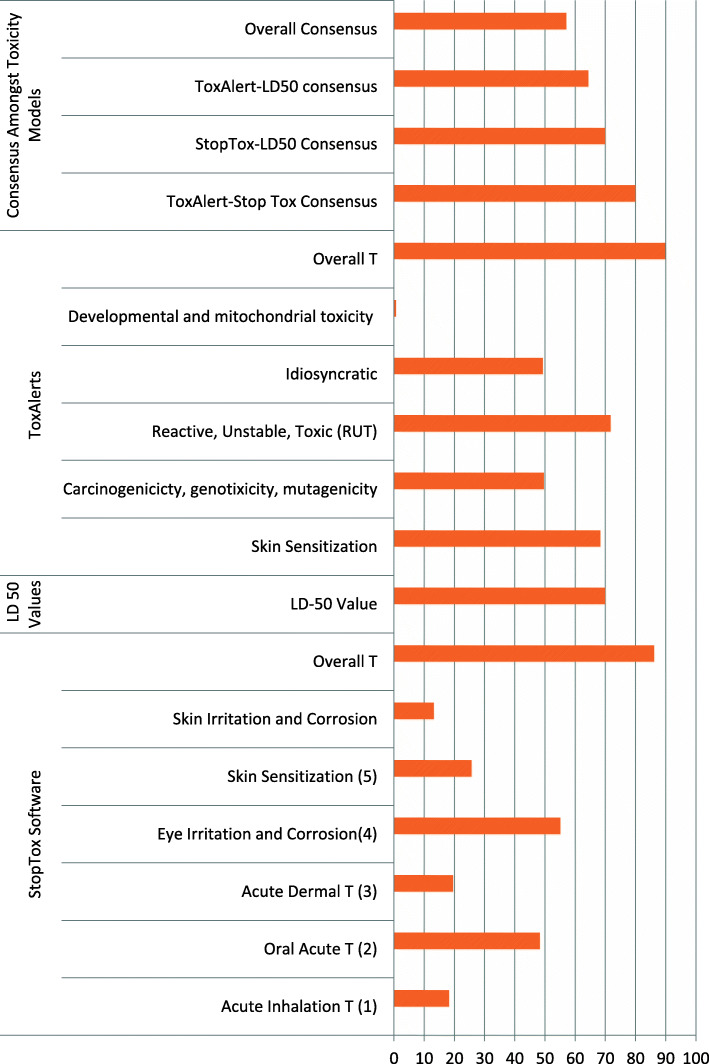
Number and percentages of drugs with potential toxicities

The drugs were analyzed based on the type of toxicity predicted in each model, the number of toxicities each drug had the potential of causing in the patients as well as the consensus between any two models and amongst all the models. Figure 2 shows the predicted toxicities in each model. In the StopTox model, 18% (54/296) of the drugs had the potential of causing acute inhalation toxicity. 48% (143/296) of the drugs had the potential of causing acute oral toxicity, while 20% (58/296) drugs had the potential of causing acute dermal toxicity. On the other hand, 55% (163/296) drugs had the potential of causing eye irritation and corrosion, while 26% (76/296) had the potential of causing skin sensitization. Furthermore, 13% (39/296) had the potential of causing skin irritation and corrosion, while 14% (42/296) had none of the indicated toxicities.

40% (117/296) of the drugs had 1 toxicity only; 22% (65/296) drugs had 2 toxicities; 9% (28/296) of drugs had 3 toxicities; 10% (29/296) had 4 toxicities; 4% (13/296) drugs had 5 toxicities and 1% (3/296) had all the 6 toxicities. Figure 2 below shows a summary of the toxicities cited above

In ToxAlerts, 1% (2/294) of the drugs showed potential of causing developmental and mitochondrial toxicity and 49% (145/294) drugs had potential of causing idiosyncratic adverse drug reactions. 50% (146/294) of the drugs were found to be potentially genotoxic, mutagenic or carcinogenic and 68% (201/294) were found to be skin sensitive. 72% (211/294) were found to be potentially reactive, unstable and toxic. 90% (264/294) of the drugs had the potential of causing at least one toxicity.

In case of LD-50 values based toxicity evaluation, 70% (205/293) of the drugs were potentially toxic (Fig. 2).

The three toxicity prediction models were evaluated for consensus, in pairs and all of them combined. Descriptive analysis showed that the highest consensus was observed between ToxAlerts and StopTox (80%, 235/294) followed by StopTox and LD-50 values (70%, 205/293) and ToxAlert-LD-50 values (64%, 188/292). The overall consensus for all the models was 57% (169/296) (Fig. 2).

**Fig. 3:**
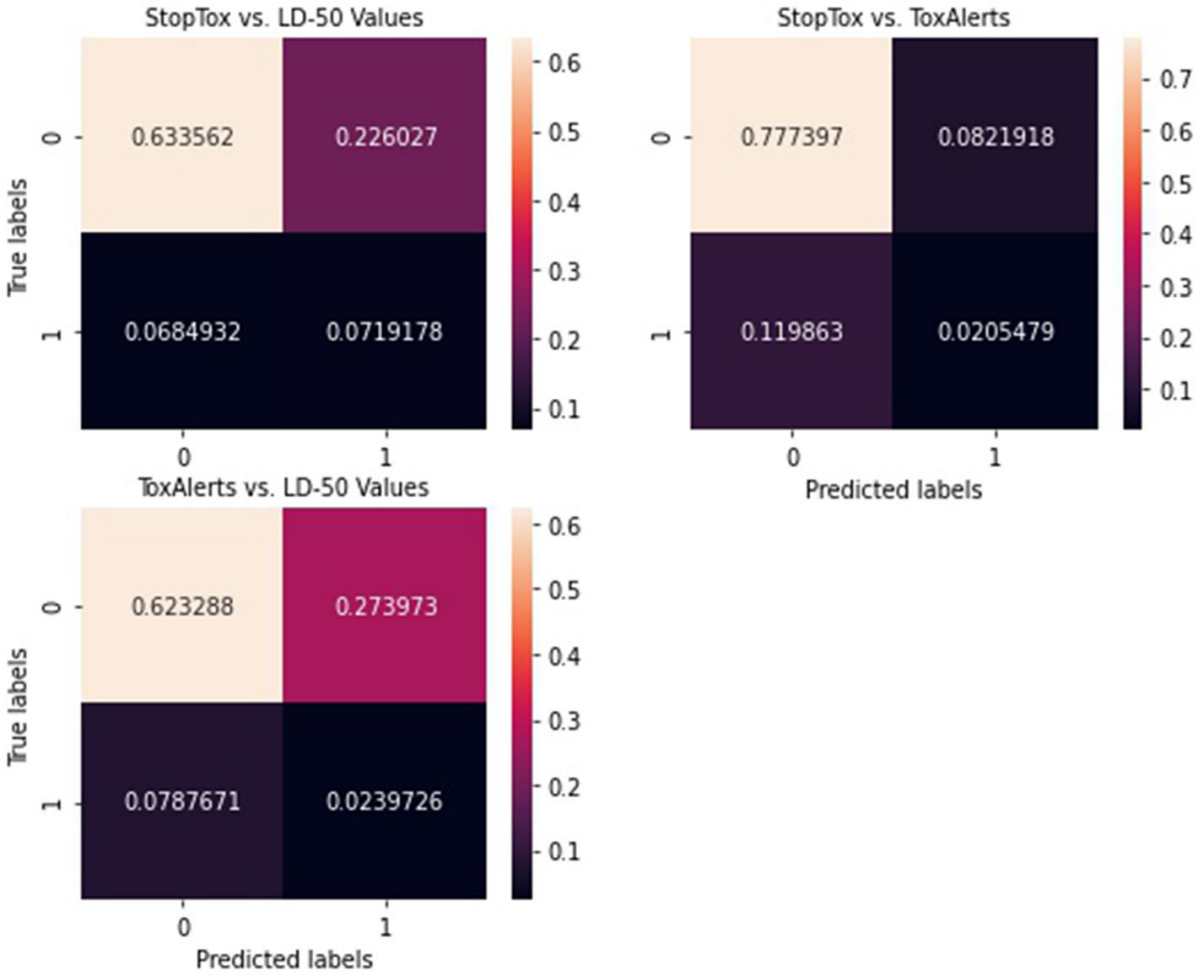
Confusion matrix for StopTox, ToxAlerts and LD-50 values

Consensus measure based on confusion matrix between every two models showed that there was consensus in the tests of every pair of the models. Firstly, confusion matrix showed that the proportion of true positives and true negatives, ie, the cases where all the models found that the drugs were non-toxic and/or toxic (consensus) was higher than the cases where there were different results where by one model showed toxicity and the other showed non-toxic (non-consensus). Secondly, the confusion matrix showed that there was greater consensus between StopTox and ToxAlerts (0.78, 0.02; meaning 80% consensus) than StopTox and LD-50 values (0.63, 0.07; meaning 70% consensus) as well as ToxAlerts and LD-50 (0.62, 0.02; meaning 64%). Figure 3 above summarises the confusion matrix results for the three models.

The results of Fig. 2 showed that the overall consensus was over 50% amongst all the three models. So this agreement was evaluated to find out if it was significant statistically using bimonial test. The results of the binomial test showed that the *p*-value was *p* = 0.0059, which was much lower than the selected *a* = 0.05. Thus, these results showed that the agreement observed in the three models was statistically significant.

## Discussion

The results show that the majority of the drugs have the potential to cause systemic toxicities (70–90%) (Fig. 2; Table 1; Supplementary Table 1). However, it is also reported that the occurrence of the drug-induced toxicities depends on many factors such as dosage, metabolites reactivity, structural alert metabolism, competition for detoxification pathways, and individual differences between patients [3, 22–24]. The results of the analyses have also shown that the majority of the drugs (72%) were potentially reactive, unstable or toxic, and 70% of them were also potentially toxic at normal treatment concentrations, based on their doses or LD-50 values evaluation (Supplementary Table 1, Fig. 2). The activity of a structural alert depends on the molecule to which it is attached, as illustrated in the thiophene structural alert in methapyrilene and eprosartan, where in methapyrilene, the structural alert undergoes bioactivation but the same thiophene is not activated in eprosartan. Methapyrilene was withdrawn from the market because of hepatotoxicity while eprosartan is safe and is prescribed for hypertension [25, 26]. In the USA, 50% of the 200 most frequently prescribed drugs were found to have at least one structural alert, yet the majority of the drugs with structural alerts were not associated with IADRs. This shows that structural alerts do not predict metabolism and toxicity adequately. Therefore, some medicines may be designated as safe or unsafe when actually it is not true [3, 22]. The drug-induced toxicities caused by structural alerts and reactive metabolites may be caused by either covalent interactions or noncovalent interactions with cellular macromolecules such as DNA, proteins and lipids [27], but in many cases their exact mechanism is not known [3, 22]. About 78–86% of drugs, which is a substantial proportion of drugs used, are found to have structural alerts linked to particular organ toxicity or unspecified adverse drug reactions, most of which are idiosyncratic adverse drug reactions (IADRs). It is also widely reported that a substantial proportion (62–69%) of drugs in use have the potential to form reactive metabolites. However, not all structural alerts lead to toxicities as there are some molecules that contain structural alerts but never generate toxic effects [22].

The results of our study are in agreement with a study by Liu et al. [10] and Pizzo et al. [28] in which they found some structural alerts that had the potential for liver induced toxicities and the drugs that contained such structural alerts. The structural alerts that the studies found were compared with the structures of the drugs in the MEML and it was found that indeed some drugs had toxicity potential. When these drugs were fed into the StopTox software, it was confirmed that these drugs had at least one predicted toxicity (Fig. 2).

Predicting toxicity too often maybe regarded as a safe position that reduces the risk of exposing patients to dangerous drugs. However, it also has the potential to stop the development of useful drugs that are not actually toxic in clinical use. Prediction *in-silico* is currently a useful tool but cannot replace in-vivo or in-vitro toxicity testing.

This study also showed that there was generally consensus between the three models for the majority of the medicines studied (Figs. 2 and 3). However, greater consensus was noted between Stoptox and the other two models. This could be attributed to the fact that the StopTox incorporates all the parameters used for the development of the other models, i.e., functional groups or structural alerts for ToxAlerts and doses in case of LD-50 values. The lower consensus between LD-50 values and ToxAlerts could be attributed to the limited overlap between the two models, i.e., LD-50 uses only concentration while ToxAlerts uses largely the functional groups of structural alerts although both parameters play significant role in the two models, explaining the siginificant overlap observed in the two models. The differences in the overlap amongst the models are worrying as they bring an issue of choice of the model to be used. Making a choice without substantial evidence could be challenging. In the literature, there are no guidelines yet for choosing a model for predicting toxicity of the drugs. Furthermore, although the two programmes were compared, it was challenging to attribute their similaries or differences to their limitations or strengths because they mostly had different endpoints for the toxicity predictions, which makes comparison a little inconclusive. Furthermore, it is challenging to make a conclusive decision based on the overall consensus because it is still on the lower side despite being over 50%.

Therefore, it is important to conduct further studies in Malawi starting with clinical data to find out if the indicated toxicities indeed occur or not for the medicines designated as toxic or not. Furthermore, it is also important to find out if the underlying conditions suggestive of the toxicity occurrence are also present or absent for the medicines found to be toxic or non-toxic. This would act as an evaluation of the clinical application of the software as well as the provision of further data for the development of the software so that it can be as reliable as possible for future use for clinical, research and regulatory purposes. However, the results can still be useful now as they would give a guided identification of the targeted toxicities for the health care providers, which may enhance the patients’ safety. Furthermore, either the models should be improved to minimize the discrepancies shown in the medicines analysis, or guidelines should be developed for use of each of the models. Therefore, there is need for further development of the models for them to be able to replace animal studies whilst producing significantly reliable toxicological screening computer generated data or information.

### Limitations

This study was not spared of some difficulties. One of the limitations is that there were some difficulties in the identification of the structural alerts manually and this was minimised by using software that is designed to identify the structural alerts. Another limitation is that full interpretation of the results from software relied on US partners that we collaborated with and they have not yet given us the full interpretation especially on the differences of the colours of the highlighted functional groups. However, even without this information, we were able to get the results and interpret their meanings by the software. The use of LD-50 value ranges might use some values that were not necessarily the oral LD-50 values, and that might have affected the categorization of the medicines as toxic when they are not.

## Conclusions

The study has shown that most of the drugs in the MEML List have the potential for drug-induced toxicity. This being the case, physicians, pharmacists, and nurses should be alert when prescribing, dispensing, and administering these drugs so that the well-being of the patients is safeguarded. However, it should be noted that not all the drugs with potential for toxicities will ultimately cause drug-induced toxicities. This is because the occurrence of toxicities depends on many factors, such as concentration and exposure. More studies with a variety of software that extend to human studies should be conducted to have conclusive results. Clinical studies are also needed to provide data for comparison.

## Supplementary Information

**Additional file 1: Supplementary Table 1**. Screening results of the Malawi Essential Medicines and Reactive Metabolites

## Data Availability

All the data related to the study have been included.

## Abbreviations

MEML: Malawi Essential Medicines List
SAs: Structural alerts
COMREC: College of Medicine Research and Ethics Committee
SMARTS: Smiles Arbitrary Target Specification
DNA: Deoxyribonucleic acid
RNA: Ribonucleic acid
SMILES: Simplified Molecular Input Line Entry Specification
COM: College of Medicine
QSAR: Quantitative Structural Activity Relationship
