# Supplementary material for: In-silico evaluation of Malawi essential medicines and reactive metabolites for potential drug-induced toxicities
Source: BMC Pharmacol Toxicol. 2021 Jun 16;22:36. doi: 10.1186/s40360-021-00499-6 (PMC8207713; doi:10.1186/s40360-021-00499-6)
Supplement: Supplementary file 1 — Additional file 1: Supplementary Table 1. Screening results of the Malawi Essential Medicines and Reactive Metabolites [file 40360_2021_499_MOESM1_ESM.docx]

***In-Silico* Evaluation of Malawi Essential Medicines and Reactive Metabolites for Potential Drug-Induced Toxicities**

Ibrahim Chikowe^1*^, Alfred Chipanda Phiri^1^, Kirios Patrick Mbewe^1^, Dunstan Matekenya^2^

^1^Pharmacy Department, College of Medicine, University of Malawi.

^2^The World Bank Group, DECDG, Washington DC.

*Corresponding Author: [chikoweib@yahoo.co.uk](mailto:chikoweib@yahoo.co.uk), [ichikowe@medcol.mw](mailto:ichikowe@medcol.mw).

Supplementary Table 1: Screening results of the Malawi Essential Medicines and Reactive Metabolites

| **Drug Name** | **StopTox Software** | | | | | | | **LD 50 Values** | | | **ToxAlerts** | | | | | | **Consensus Amongst Toxicity Models** | | | |
| --- | --- | --- | --- | --- | --- | --- | --- | --- | --- | --- | --- | --- | --- | --- | --- | --- | --- | --- | --- | --- |
|  | Acute Inhalation T (1) | Oral Acute T (2) | Acute Dermal T (3) | Eye Irritation and Corrosion(4) | Skin Sensitization(5) | Skin Irritation and Corrosion | Overall T | LD-50 Value | T Range | Overall T | Skin Sensitization | Carcinogenicicty, genotixicity, mutagenicity | RUT | Idiosyncratic | Dev, mitochond | Overall T | ToxAlert-Stop Tox Consensus | StopTox-LD50 Consensus | ToxAlert-LD50 consensus | Overall Consensus |
| Acetazolamide | NT | NT | NT | T | NS | -ve | T | 54-4300 | moderate to slight T | T | T | T | NT | NT | NT | T | C | C | C | C |
| Acetylsalicylic acid (Aspirin) | NT | T | NT | NT | NS | -ve | T | 0.92-7940 | Highest T to Safe | Safe | T | T | T | NT | NT | T | C | NC | NC | NC |
| 5 Fluorouracil | NT | NT | NT | T | NS | -ve | T | 230 | Moderate T | T | NT | NT | NT | NT | NT | T | C | C | C | C |
| Acetic acid | T | T | NT | T | NS | +ve | T | 1060-5620 | Slight T to Safe | Safe | NT | NT | NT | T | NT | T | C | NC | NC | NC |
| Acyclovir | NT | NT | NT | T | NS | -ve | T | 20000 | Slight T | T | T | T | NT | NT | NT | T | C | C | C | C |
| Adrenaline/Epinephrine | NT | T | T | NT | S | -ve | T | 62 | Highest T | T | T | NT | T | T | NT | T | C | C | C | C |
| Adriamycin (Doxorubicin) | NT | T | NT | T | NS | -ve | T | 21.8 | Highest T | T | T | T | T | T | NT | T | C | C | C | C |
| Albendazole | NT | NT | NT | T | NS | -ve | T | 2400 | Slight T | T | T | T | NT | NT | NT | T | C | C | C | C |
| Alcuronium chloride | NT | T | NT | NT | NS | -ve | T | 27.6 | Highest T | T | T | T | T | NT | NT | T | C | C | C | C |
| Allopurinol | NT | T | NT | T | NS | -ve | T | 10-100 | Highest to moderate T | T | NT | NT | NT | NT | NT | Safe | NC | C | NC | NC |
| Amethocaine | T | T | T | T | S | -ve | T | 300-500 | moderate T | T | T | T | T | T | NT | T | C | C | C | C |
| Amikacin | NT | NT | NT | T | NS | -ve | T | 6000 | Safe | Safe | T | NT | T | NT | NT | T | C | NC | NC | NC |
| Aminophylline | NT | NT | NT | T | NS | -ve | T | 243 | Moderate T | T | NT | NT | NT | NT | NT | Safe | NC | C | NC | NC |
| Amitriptyline | T | T | T | NT | S | +ve | T | 0.714-320 | Highest to moderate T | T | T | NT | NT | NT | NT | T | C | C | C | C |
| Amlodipine | NT | T | NT | T | NS | -ve | T | 37 | Highest T | T | T | T | T | T | T | T | C | C | C | C |
| Amodiaquine | NT | T | NT | T | S | -ve | T | 225-550 | Moderate T | T | T | T | T | T | NT | T | C | C | C | C |
| Amoxicillin | NT | NT | NT | T | NS | -ve | T | 15000 | Safe | Safe | T | NT | T | T | NT | T | C | NC | NC | NC |
| Amphotericin B | NT | NT | NT | T | NS | -ve | T | 5000 | Safe | Safe | T | NT | T | T | NT | T | C | NC | NC | NC |
| Ampicillin | NT | NT | NT | T | S | -ve | T | 50 | Highest T | T | T | NT | NT | T | NT | T | C | C | C | C |
| Anastrozole | T | T | NT | T | S | -ve | T | 45-100 | Highest to moderate T | T | NT | NT | NT | NT | NT | Safe | NC | C | NC | NC |
| Artemether | NT | NT | NT | NT | NS | +ve | T | 296-895 | Moderate to slight T | T | T | NT | T | NT | NT | T | C | C | C | C |
| Artesunate | NT | NT | NT | NT | NS | -ve | Safe | 600-900 | Slight T | T | T | NT | T | T | NT | T | NC | NC | C | NC |
| Ascorbic acid | NT | NT | NT | NT | NS | -ve | Safe | 643-11900 | slight T to safe | Safe | T | T | T | T | NT | T | NC | C | NC | NC |
| Atenolol | NT | NT | NT | NT | NS | -ve | Safe | 50-2000 | Highest to slight T | T | NT | NT | NT | NT | NT | Safe | C | NC | NC | NC |
| Atorvastatin | NT | NT | NT | T | NS | -ve | T | 5000 | Safe | Safe | T | T | T | T | NT | T | C | NC | NC | NC |
| Atovaquone | NT | T | NT | NT | NS | -ve | T | 1825 | Slight Toxicity | T | T | T | T | T | NT | T | C | C | C | C |
| Atropine | NT | T | NT | NT | NS | -ve | T | 75-500 | Moderate Toxicity | T | NT | NT | T | NT | NT | T | C | C | C | C |
| Azathioprine | NT | T | NT | T | NS | -ve | T | 400-2500 | Moderate to slight T | T | NT | T | T | T | NT | T | C | C | C | C |
| Azithromycin | NT | NT | NT | T | S | -ve | T | 2000 | Slight T | T | T | NT | T | NT | NT | T | C | C | C | C |
| Baclofen | NT | T | T | NT | S | -ve | T | 145 | Moderate T | T | T | T | NT | T | NT | T | C | C | C | C |
| Barium sulphate | NT | T | NT | T | S | +ve | T | 2000 | Slight T | T | NT | NT | T | NT | NT | T | C | C | C | C |
| Beclomethasone dipropionate | NT | NT | NT | NT | NS | -ve | T | 3750 | Slight T | T | T | T | T | T | NT | T | C | C | C | C |
| Bendrofluazide | NT | NT | NT | T | NS | -ve | T | 4800-10000 | slight T to safe | Safe | T | T | T | NT | NT | T | C | NC | NC | NC |
| Benzathine penicillin/Benzylpenicillin | NT | NT | NT | T | S | -ve | T | 8900 mg/kg | Safe | Safe | T | NT | NT | T | NT | T | C | NC | NC | NC |
| Benzhexol | T | T | NT | NT | NS | -ve | T | 30-1630 | Highest to slight T | T | NT | NT | NT | NT | NT | Safe | NC | C | NC | NC |
| Benzoic acid | NT | NT | NT | T | NS | -ve | T | 1250-5000 | Slight T to Safe | Safe | NT | NT | NT | T | NT | T | C | NC | NC | NC |
| Benzoyl peroxide | NT | NT | NT | T | S | -ve | T | 950-7710 | Slight T to safe | Safe | T | NT | T | NT | NT | T | C | NC | NC | NC |
| Benzyl benzoate | NT | T | NT | NT | S | -ve | T | 1000-22440 | Slight T to safe | Safe | T | NT | NT | NT | NT | T | C | NC | NC | NC |
| Betamethasone | NT | NT | NT | NT | NS | -ve | Safe | 1877-4500 | Slight T | T | T | T | T | NT | NT | T | NC | NC | C | NC |
| Betaxolol | NT | T | NT | NT | S | -ve | T | 350-980 | Moderate to slight T | T | NT | NT | NT | NT | NT | Safe | NC | C | NC | NC |
| Bicalutamide | NT | NT | NT | T | NS | -ve | T | 200-2000 | moderate to slight T | T | T | T | T | T | T | T | C | C | C | C |
| Bimatoprost | NT | NT | NT | T | NS | -ve | T | No data available | No data available | Undefined | NT | NT | NT | NT | NT | Safe | NC |  | NC | NC |
| Biperiden | T | T | NT | NT | NS | -ve | T | 750-760 | Moderate to slight T | T | NT | NT | NT | NT | NT | Safe | NC | C | NC | NC |
| Bisacodyl | NT | NT | NT | NT | NS | -ve | Safe | 4320 | Slight T | T | T | NT | T | NT | NT | T | NC | NC | C | NC |
| Bismuth chelate/Bismuth subsalicylate | NT | T | T | T | S | -ve | T | 2000 | Slight T | T | NT | NT | T | NT | NT | T | C | C | C | NC |
| Bismuth subgallate | NT | NT | NT | T | NS | -ve | T | 2000 | Slight T | T | T | NT | T | T | NT | T | C | C | C | C |
| Bleomycin | NT | NT | NT | T | NS | -ve | T | 50-240 | Moderate T | T | T | NT | T | NT | NT | T | C | C | C | C |
| Brimonidine | T | T | T | T | NS | -ve | T | 50- 100 | Moderate T | T | T | T | T | NT | NT | T | C | C | C | C |
| Brinzolamide | NT | T | NT | T | NS | -ve | T | 1000-2000 | Slight T | T | NT | NT | NT | T | NT | T | C | C | C | C |
| Bromocriptine | NT | NT | NT | T | NS | -ve | T | 0.81-2502 | Highest to slight T | T | T | T | T | T | NT | T | C | C | C | C |
| Bupivacaine | T | NT | NT | NT | NS | -ve | T | 6.0-43.0 | Highest T | T | T | NT | T | T | NT | T | C | C | C | C |
| Busulphan | NT | T | NT | T | S | +ve | T | 18-110 | Highest to moderate T | T | T | NT | T | NT | NT | T | C | C | C | C |
| Calcium gluconate | NT | NT | NT | NT | NS | -ve | Safe | 950 | Moderate T | T | NT | NT | T | T | NT | T | NC | NC | C | NC |
| Capreomycin | NT | NT | NT | T | NS | -ve | T | 514 | Slight T | T | T | T | T | NT | NT | T | C | C | C | C |
| Captopril | NT | NT | NT | T | S | -ve | T | 4245 | Slight T | T | T | NT | T | T | NT | T | C | C | C | C |
| Carbamazepine | NT | T | NT | NT | NS | -ve | T | 1957 | Slight T | T | T | T | T | NT | NT | T | C | C | C | C |
| Carbex/Selegiline | T | T | T | NT | S | +ve | T | 63 | Moderate T | T | NT | NT | NT | T | NT | T | C | C | C | C |
| Carbidopa | NT | NT | NT | NT | S | -ve | T | 4810 | Slight T | T | T | T | T | T | NT | T | C | C | C | C |
| Carbimazole***** | T | T | NT | T | NS | -ve | T | 300-2000 | Moderate to Slight T | T | NT | NT | NT | NT | NT | Safe | NC | C | NC | NC |
| Cefepime | NT | NT | NT | T | NS | -ve | T | 1272-2000 | Slight T | T | T | T | T | T | NT | T | C | C | C | C |
| Cefotaxime | NT | NT | NT | T | NS | -ve | T | 7000-20000 | Safe | Safe | T | T | T | T | NT | T | C | NC | NC | NC |
| Ceftazidime | NT | NT | NT | T | NS | -ve | T | 20000 | Safe | Safe | T | T | T | T | NT | T | C | NC | NC | NC |
| Ceftriaxone | NT | NT | NT | T | NS | -ve | T | 5000-10000 | Safe | safe | T | T | T | T | NT | T | C | NC | NC | NC |
| Cetirizine | NT | T | NT | NT | NS | -ve | T | 365 | Moderate T | T | T | T | T | T | NT | T | C | C | C | C |
| Cetrimide/Cetrimonium | T | NT | T | T | S | +ve | T | 410 | Moderate T | T | T | NT | T | NT | NT | T | C | C | C | C |
| Chlorhexidine | T | T | T | NT | S | -ve | T | 5000 | Slight T to safe | Safe | T | T | T | NT | NT | T | C | NC | NC | NC |
| Chlorambucil | T | T | NT | NT | NS | -ve | T | 76 | Moderate T | T | T | T | T | T | NT | T | C | C | C | C |
| Chloramphenicol | NT | NT | NT | T | S | -ve | T | 1500-2500 | Slight T | T | T | T | T | T | NT | T | C | C | C | C |
| Chlordiazepoxide | NT | T | NT | T | S | -ve | T | 537 | Slight T | T | NT | T | T | NT | NT | T | C | C | C | C |
| Chloroquine | T | T | T | NT | S | -ve | T | 392 | Moderate T | T | T | T | T | NT | NT | T | C | C | C | C |
| Chlorpheniramine maleate | T | T | T | NT | NS | +ve | T | 130-1600 | Moderate to slight T | T | T | T | NT | NT | NT | T | C | C | C | C |
| Chlorpromazine | T | T | T | NT | S | +ve | T | 142 | Moderate T | T | T | T | T | T | NT | T | C | C | C | C |
| Cimetidine | T | NT | T | T | NS | -ve | T | 5000 | Slight T to safe | Safe | NT | NT | T | NT | NT | T | C | NC | NC | NC |
| Ciprofloxacin | NT | NT | NT | T | NS | -ve | T | 2000 | Slight T | T | T | T | T | T | NT | T | C | C | C | C |
| Clarithromycin | NT | T | NT | T | NS | -ve | T | 1270 | Slight T | T | T | NT | T | NT | NT | T | C | C | C | C |
| Clavulanic acid | NT | NT | NT | T | NS | -ve | T | 2000-7936 | Slight T to safe | Safe | T | T | NT | T | NT | T | C | NC | NC | NC |
| Clindamycin | NT | NT | NT | T | NS | -ve | T | 2540-2190 | Slight T | T | T | T | T | T | NT | T | C | C | C | C |
| Clobetasol propionate | NT | NT | NT | NT | NS | -ve | Safe | 3000 | Slight T | T | T | T | T | T | NT | T | NC | NC | C | NC |
| Clofazimine | T | NT | T | T | S | -ve | T | 5000-8400 | Safe | Safe | T | T | T | T | NT | T | C | NC | NC | NC |
| Clomiphene citrate | T | NT | T | NT | NS | -ve | T | 1700-5750 | Slight T to safe | Safe | T | NT | NT | NT | NT | T | C | NC | NC | NC |
| Clotrimazole | T | T | T | T | S | +ve | T | 108-10000 | Moderate T to safe | Safe | NT | T | T | NT | NT | T | C | NC | NC | NC |
| Cloxacillin | NT | NT | NT | T | S | -ve | T | 1660-5000 | Slight T to safe | Safe | T | T | T | T | NT | T | C | NC | NC | NC |
| Codeine phosphate | NT | T | NT | NT | NS | -ve | T | 85-427 | Moderate T | T | T | T | NT | NT | NT | T | C | C | C | C |
| Colchicine | NT | NT | NT | NT | NS | -ve | Safe | 5.87 | Highest T | T | T | NT | NT | NT | NT | T | NC | NC | C | NC |
| Cyclizine | T | T | T | NT | S | -ve | T | 58-147 | Moderate T | T | NT | NT | NT | NT | NT | Safe | NC | C | NC | NC |
| Cyclopentolate | NT | T | NT | NT | NS | -ve | T | 960-4000 | Slight T | T | T | NT | T | NT | NT | T | C | C | C | C |
| Cyclophosphamide | NT | T | T | T | NS | +ve | T | 100 | Moderate T | T | T | T | T | T | NT | T | C | C | C | C |
| Cycloserine | NT | NT | NT | T | NS | -ve | T | 5000-5290 | Safe | Safe | NT | NT | NT | NT | NT | Safe | NC | NC | C | NC |
| Cyclosporine | NT | NT | NT | T | NS | -ve | T | 1450-1480 | Slight T | T | T | NT | T | NT | NT | T | C | C | C | C |
| Dapsone | NT | T | NT | NT | NS | -ve | T | 250-2000 | Moderate to slight T | T | T | T | T | NT | NT | T | C | C | C | C |
| Desferrioxamine | NT | NT | NT | T | S | -ve | T | 340-3000 | Moderate to slight T | T | T | T | T | NT | NT | T | C | C | C | C |
| Dexamethasone | NT | NT | NT | NT | NS | -ve | Safe | 794-6500 | Moderate T to safe | Safe | T | T | T | T | NT | T | NC | C | NC | NC |
| Diazepam | T | T | NT | T | S | -ve | T | 249-1070 | Moderate to slight T | T | T | T | T | T | NT | T | C | C | C | C |
| Diclofenac sodium | NT | T | NT | NT | NS | -ve | T | 53-3200 | Moderate toslight T | T | T | NT | T | T | NT | T | C | C | C | C |
| Didanosine | NT | NT | NT | T | NS | -ve | T | 2000 | Slight T | T | NT | NT | NT | NT | NT | Safe | NC | C | NC | NC |
| Digoxin | NT | T | NT | NT | NS | -ve | T | 28 | Highest T | T | T | T | T | T | NT | T | C | C | C | C |
| Dihydrocodeine tartrate | NT | T | NT | NT | NS | -ve | T | 359 | Moderate T | T | T | T | NT | NT | NT | T | C | C | C | C |
| Dinoprostone | NT | T | NT | NT | S | -ve | T | 500-750 | Slight T | T | T | NT | T | T | NT | T | C | C | C | C |
| Dithranol | NT | NT | T | T | S | -ve | T | 359 | Moderate T | T | T | NT | T | NT | NT | T | C | C | C | C |
| Dorzolamide | NT | T | NT | T | NS | -ve | T | 1320-2000 | Slight T | T | NT | NT | NT | T | NT | T | C | C | C | C |
| Doxycycline | NT | NT | NT | T | NS | -ve | T | 2000 | Slight T | T | T | T | T | T | NT | T | C | C | C | C |
| Econazole | NT | T | NT | T | NS | -ve | T | 160-668 | Moderate T | T | NT | T | NT | NT | NT | T | C | C | C | C |
| Edrophonium chloride | T | T | NT | NT | S | -ve | T | 30-50 | High T | T | T | NT | T | NT | NT | T | C | C | C | C |
| Efavirenz | NT | T | T | NT | NS | -ve | T | 30-52 | Highest to moderate T | T | T | T | T | T | NT | T | C | C | C | C |
| Emedastine | T | T | NT | NT | NS | -ve | T | 628-1854 | Slight T | T | T | T | NT | T | NT | T | C | C | C | C |
| Enalapril | NT | NT | NT | T | NS | -ve | T | 2973 | Slight T | T |  |  |  |  |  |  |  | C |  | C |
| Ergometrine maleate | NT | T | NT | T | NS | -ve | T | 8.3-93 | Highest to moderate T | T | T | NT | T | T | NT | T | C | C | C | C |
| Erythromycin | NT | NT | NT | NT | NS | -ve | Safe | 2580-9272 | Slight T to safe | Safe | T | NT | T | NT | NT | T | NC | C | NC | NC |
| Estradiol/Estrogen | NT | NT | NT | NT | NS | -ve | Safe | 960-5000 | Slight T to safe | Safe | NT | NT | T | T | NT | T | NC | C | NC | NC |
| Ethambutol | T | NT | NT | NT | NS | -ve | T | 998 | Slight T | T | T | NT | NT | NT | NT | T | C | C | C | C |
| Ethinylestradiol | NT | NT | NT | NT | NS | -ve | Safe | 960 | Slight T | T | NT | NT | T | T | NT | T | NC | NC | C | NC |
| Ethionamide | T | T | T | NT | NS | -ve | T | 1320 | Slight T | T | NT | T | T | T | NT | T | C | C | C | C |
| Ethosuximide | T | T | NT | T | NS | -ve | T | 1820 | Slight T | T | T | NT | NT | NT | NT | T | C | C | C | C |
| Ethyl chloride | NT | T | T | T | NS | +ve | T | 1500 | Slight T | T | T | T | T | T | NT | T | C | C | C | C |
| Ferrous sulphate | NT | T | NT | T | S | +ve | T | 13-2625 | Highest to slight T | T | NT | NT | T | NT | NT | T | C | C | C | C |
| Feruxomide or Iron (III) Oxide | T | T | NT | T | NS | +ve | T | 5000 | Safe | Safe | NT | NT | T | NT | NT | T | C | NC | NC | NC |
| Flucloxacillin | NT | NT | NT | T | S | -ve | T | 680-11000 | Moderate T to Safe | Safe | T | NT | T | T | NT | T | C | NC | NC | NC |
| Fluconazole | NT | NT | NT | T | S | -ve | T | 1271 | Slight T | T | NT | NT | NT | NT | NT | Safe | NC | C | NC | NC |
| Fludrocortisone | NT | NT | NT | NT | NS | -ve | Safe | 1000 | Slight T | T | T | T | T | T | NT | T | NC | NC | C | NC |
| Fluocinolone acetonide | NT | T | T | NT | NS | -ve | T | 4000 | Slight T | T | T | T | T | T | NT | T | C | C | C | C |
| Fluorescein sodium | NT | NT | T | T | S | -ve | T | 6721 | Safe | Safe | T | NT | T | T | NT | T | C | NC | NC | NC |
| Fluorometholone | NT | T | NT | NT | NS | -ve | T | 234 | Moderate T | T | T | T | T | T | NT | T | C | C | C | C |
| Fluoxetine | NT | T | NT | NT | S | -ve | T | 825 | Slight T | T | T | NT | T | NT | NT | T | C | C | C | C |
| Fluphenazine | NT | T | NT | T | NS | +ve | T | 89-640 | Moderate to Slight T | T | NT | T | T | T | NT | T | C | C | C | C |
| Folic acid | NT | NT | NT | T | NS | -ve | T | 85-500 | Moderate T | T | T | T | T | T | NT | T | C | C | C | C |
| Foscarnet | NT | NT | NT | NT | NS | +ve | T | 2000 | Slight T | T | NT | NT | T | NT | NT | T | C | C | C | C |
| Furosemide | NT | NT | NT | T | NS | -ve | T | 800-4600 | Slight T | T | T | T | T | T | NT | T | C | C | C | C |
| Gabapentine | NT | NT | NT | NT | NS | -ve | Safe | 8000 | Safe | Safe | T | NT | NT | T | NT | T | NC | C | NC | NC |
| Ganciclovir | NT | NT | NT | T | NS | -ve | T | 150-2000 | Moderate to slight T | T | T | T | NT | NT | NT | T | C | C | C | C |
| Gastrografin/Diatrizoate | NT | NT | NT | T | NS | -ve | T | 11300-44504 | Safe | Safe | T | T | T | T | NT | T | C | NC | NC | NC |
| Gentamicin | NT | NT | NT | T | NS | -ve | T | 52-6600 | Moderate to safe | Safe | T | NT | T | NT | NT | T | C | NC | NC | NC |
| Gentian violet | T | T | T | NT | S | -ve | T | 420 | Moderate T | T | T | T | T | T | NT | T | C | C | C | C |
| Glibenclamide | NT | NT | NT | T | NS | -ve | T | 1500-20000 | Slight toicity to safe | Safe | T | T | T | T | NT | T | C | NC | NC | NC |
| Gliclazide | NT | NT | NT | T | NS | -ve | T | 3000 | Slight T | T | T | T | T | NT | NT | T | C | C | C | C |
| Glycerine/Glycerol | NT | NT | NT | NT | NS | -ve | Safe | 4090-12600 | Slight T to safe | Safe | NT | NT | NT | NT | NT | Safe | C | C | C | C |
| Glyceryl trinitrate | NT | T | T | T | S | -ve | T | 23.2-9560 | Highest T to safe | Safe | NT | NT | T | NT | NT | T | C | NC | NC | NC |
| Griseofulvin | NT | NT | T | NT | NS | -ve | T | 10000 | Safe | Safe | T | T | T | T | NT | T | C | NC | NC | NC |
| Halofantrine | T | NT | NT | NT | S | -ve | T | 154 | Moderate T | T | T | T | T | NT | NT | T | C | C | C | C |
| Haloperidol | NT | T | NT | NT | NS | -ve | T | 71-128 | Moderate T | T | T | T | NT | T | NT | T | C | C | C | C |
| Hydralazine HCL | NT | T | T | NT | S | -ve | T | 90-187 | Moderate T | T | T | T | T | NT | NT | T | C | C | C | C |
| Hydrochlorothiazide | NT | NT | NT | T | NS | -ve | T | 2750-10000 | Slight T to safe | Safe | T | T | T | NT | NT | T | C | NC | NC | NC |
| Hydrocortisone acetate | NT | NT | NT | NT | NS | -ve | Safe | 2300-5000 | Slight T to safe | Safe | T | T | T | T | NT | T | NC | C | NC | NC |
| Hydrogen peroxide | T | T | NT | NT | NS | +ve | T | 75-4060 | Moderate to slight T | T | NT | T | T | NT | NT | T | C | C | C | C |
| Hydroxocobalamin | NT | NT | NT | T | NS | -ve | T | 720-1200 | Slight T | T | NT | NT | T | NT | NT | T | C | C | C | C |
| Hydroxyurea | NT | NT | NT | NT | NS | -ve | Safe | 5760-7330 | Safe | Safe | NT | T | T | NT | NT | T | NC | C | NC | NC |
| Hyoscine butylbromide/Butylscopolamine | NT | T | NT | T | NS | -ve | T | 1040 | Slight T | T | T | T | T | NT | NT | T | C | C | C | C |
| Ibuprofen | NT | NT | T | NT | NS | -ve | T | 495-1600 | Moderate to slight T | T | NT | NT | NT | T | NT | T | C | C | C | C |
| Idoxuridine | NT | NT | NT | T | NS | -ve | T | 3080 | Slight T | T | NT | NT | T | NT | NT | T | C | C | C | C |
| Ifosfamide | NT | T | T | T | NS | +ve | T | 143-1005 | Moderate to slight T | T | T | T | T | T | NT | T | C | C | C | C |
| Imipenem | NT | NT | NT | T | NS | -ve | T | 5000 | Safe | Safe | T | NT | T | T | NT | T | C | NC | NC | NC |
| Indomethacin | NT | T | NT | T | NS | -ve | T | 2.42-50 | Highest T | T | T | T | T | T | NT | T | C | C | C | C |
| Iodine | NT | T | T | T | NS | +ve | T | 1425-14000 | Slight T to safe | Safe | NT | NT | T | NT | NT | T | C | NC | NC | NC |
| Isoniazid | NT | T | NT | NT | NS | -ve | T | 100-1250 | Moderate to slight T | T | NT | T | T | NT | NT | T | C | C | C | C |
| Isosorbide dinitrate | NT | NT | T | T | S | -ve | T | 747-3000 | Slight T | T | NT | NT | T | NT | NT | T | C | C | C | C |
| Ivermectin | NT | NT | T | NT | NS | -ve | T | 10-29.5 | Highest T | T | NT | NT | T | NT | NT | T | C | C | C | C |
| Kanamycin | NT | NT | NT | T | NS | -ve | T | 3000-17500 | Slight T to safe | Safe | T | NT | T | NT | NT | T | C | NC | NC | NC |
| Ketoconazole | NT | T | NT | T | NS | -ve | T | 166 | Moderate T | T | T | T | T | NT | NT | T | C | C | C | C |
| Ketotifen | NT | T | NT | NT | NS | -ve | T | 360-820 | Moderate toslight T | T | T | NT | T | T | NT | T | C | C | C | C |
| Lactulose | NT | NT | NT | NT | NS | -ve | Safe | 18160 | Safe | Safe | T | NT | T | NT | NT | T | NC | C | NC | NC |
| Lamivudine | NT | NT | NT | T | NS | -ve | T | 2000-4000 | Slight T | T | T | T | NT | NT | NT | T | C | C | C | C |
| Leucovorin | NT | NT | NT | T | NS | -ve | T | 8000 | Safe | Safe | T | T | NT | NT | NT | T | C | NC | NC | NC |
| Levodopa | NT | NT | NT | NT | NS | -ve | Safe | 1780 | Slight T | T | T | NT | T | T | NT | T | NC | NC | C | NC |
| Levonorgestrel | NT | NT | NT | NT | NS | -ve | Safe | 5000 | Safe | Safe | T | T | T | T | NT | T | NC | C | NC | NC |
| Levothyroxine | NT | NT | NT | NT | NS | -ve | Safe | 20 | Highest T | T | NT | NT | T | T | NT | T | NC | NC | C | NC |
| Lidocaine | T | T | T | T | NS | -ve | T | 159-773 | Moderate to slight T | T | T | NT | T | T | NT | T | C | C | C | C |
| Lindane | T | T | T | NT | NS | +ve | T | 76-190 | Moderate T | T | T | T | T | T | NT | T | C | C | C | C |
| Loperamide | NT | T | NT | NT | NS | -ve | T | 98-105 | Moderate T | T | NT | T | NT | T | NT | T | C | C | C | C |
| Lorazepam | NT | NT | NT | NT | NS | -ve | Safe | 1850-4500 | Slight T | T | T | T | T | T | NT | T | NC | NC | C | NC |
| Lumefantrine | T | T | T | T | S | -ve | T | 10000 | Safe | Safe | T | NT | NT | NT | NT | T | C | NC | NC | NC |
| Lumigan (Bimatroprost) | NT | NT | NT | T | NS | -ve | T | 900-1190 | Slight T | T | NT | NT | NT | NT | NT | Safe | NC | C | NC | NC |
| Magnesium sulphate | NT | T | NT | T | S | +ve | T | 1200-2000 | Slight T | T | NT | NT | T | NT | NT | T | C | C | C | C |
| Magnesium trisilicate | NT | NT | T | NT | S | +ve | T | No data available | No data available | No data available | NT | NT | T | NT | NT | T | C |  |  | C |
| Magnevist/Gadopentetic acid | NT | NT | NT | NT | NS | -ve | Safe | 2727.8-8183.4 | Slight T to safe | Safe | NT | NT | T | T | NT | T | NC | C | NC | NC |
| Mannitol | NT | NT | NT | NT | NS | -ve | Safe | 13500-22000 | Safe | Safe | NT | NT | NT | NT | NT | Safe | C | C | C | C |
| Mebendazole | NT | T | NT | T | NS | -ve | T | 620-714 | Slight T | T | T | T | NT | NT | NT | T | C | C | C | C |
| Medroxyprogesterone acetate | NT | NT | NT | NT | NS | -ve | Safe | 900-16000 | Slight T to safe | Safe | T | T | T | T | NT | T | NC | C | NC | NC |
| Mefenamic acid | NT | T | NT | NT | NS | -ve | T | 740 | Slight T | T | T | NT | T | T | NT | T | C | C | C | C |
| Mefloquine | NT | T | NT | NT | NS | -ve | T | 880 | Slight T | T | NT | NT | T | NT | NT | T | C | C | C | C |
| Melarsoprol | NT | T | NT | NT | NS | -ve | T | 44.6 | Highest T | T | T | T | T | NT | NT | T | C | NC | NC | NC |
| Melphalan | NT | T | NT | NT | NS | -ve | T | 11.2 | Highest T | T | T | T | T | T | NT | T | C | C | C | C |
| Menthol | NT | NT | NT | NT | NS | -ve | Safe | 940-5000 | Slight T to safe | Safe | NT | NT | NT | NT | NT | Safe | C | C | C | C |
| Meropenem | NT | NT | NT | T | NS | -ve | T | 5000 | Safe | Safe | T | NT | T | T | NT | T | C | NC | NC | NC |
| Metformin | T | T | T | NT | NS | -ve | T | 225-1450 | Moderate to slight T | T | T | NT | T | NT | NT | T | C | C | C | C |
| Methotrexate | NT | T | NT | T | NS | -ve | T | 135-146 | Moderate T | T | T | T | T | T | NT | T | C | C | C | C |
| Methyldopa | NT | NT | NT | NT | S | -ve | T | 1500-5000 | Slight T to safe | Safe | T | NT | T | T | NT | T | C | NC | NC | NC |
| Methylprednisolone | NT | NT | NT | NT | NS | -ve | Safe | 100-4000 | Moderate to Slight T | T | T | T | T | T | NT | T | NC | NC | C | NC |
| Metoclopramide | NT | T | NT | T | NS | -ve | T | 750 | Slight T | T | T | T | T | T | NT | T | C | C | C | C |
| Metolazone | NT | NT | NT | T | NS | -ve | T | 5000 | Safe | Safe | T | T | T | NT | NT | T | C | NC | NC | NC |
| Metronidazole | NT | NT | NT | T | S | -ve | T | 3000-5000 | Slight T to safe | Safe | NT | T | T | T | NT | T | C | NC | NC | NC |
| Miconazole | NT | T | NT | T | S | -ve | T | 500-550 | Slight T | T | NT | NT | T | NT | NT | T | C | C | C | C |
| Misoprostol | NT | T | NT | NT | NS | -ve | T | 27-81 | Highest to moderate T | T | T | NT | T | NT | NT | T | C | C | C | C |
| Morphine sulphate | NT | T | NT | NT | NS | -ve | T | 461 | Moderate T | T | T | T | T | T | NT | T | C | C | C | C |
| Moxifloxacin | NT | NT | NT | T | NS | -ve | T | 100 | Moderate T | T | NT | T | T | T | NT | T | C | C | C | C |
| Mycophenolate mofetil | NT | T | NT | T | S | -ve | T | 250-4000 | Moderate to slight T | T | T | NT | T | T | NT | T | C | C | C | C |
| N-acetylcysteine | NT | NT | NT | T | NS | -ve | T | 400-5050 | Moderate T to Safe | Safe | T | NT | T | T | NT | T | C | NC | NC | NC |
| Nalidixic acid | NT | T | NT | T | NS | -ve | T | 572-2040 | Slight T | T | NT | NT | NT | T | NT | T | C | C | C | C |
| Naloxone HCL | NT | T | NT | NT | S | -ve | T | 109-150 | Moderate T | T | T | T | T | T | NT | T | C | C | C | C |
| Natamycin | NT | NT | NT | T | NS | -ve | T | 2730 | Slight T | T | T | T | T | T | NT | T | C | C | C | C |
| Nedocromil sodium | NT | NT | NT | T | NS | -ve | T | No available data | No available data | No available data | NT | T | NT | T | NT | T | C |  |  | C |
| Neostigmine methylsulphate | NT | T | T | T | NS | -ve | T | 0.3-0.54 | Highest T | T | T | T | T | NT | NT | T | C | C | C | C |
| Nevirapine | NT | T | NT | NT | NS | -ve | T | 502-2000 | Slight T | T | T | T | NT | NT | NT | T | C | C | C | C |
| Niclosamide | NT | T | NT | NT | S | -ve | T | 2500-5000 | Slight T to safe | Safe | T | T | T | T | NT | T | C | NC | NC | NC |
| Nicotinamide | NT | NT | NT | NT | NS | -ve | Safe | 2500-3500 | Slight T | T | NT | NT | NT | NT | NT | Safe | C | NC | NC | NC |
| Nifedipine | T | T | NT | T | NS | -ve | T | 202-1022 | Moderate to slight T | T | T | T | T | T | NT | T | C | C | C | C |
| Nimodipine | NT | NT | NT | T | NS | -ve | T | 2378 | Slight T | T | T | T | T | T | NT | T | C | C | C | C |
| Nitrate | T | T | T | T | S | +ve | T | 1600–9000 | Slight T to Safe | Safe | NT | NT | T | NT | NT | T | C | NC | NC | NC |
| Nitrofurantoin | NT | T | NT | T | S | -ve | T | 604 | Slight T | T | T | T | T | T | NT | T | C | C | C | C |
| Norethisterone | NT | NT | NT | NT | NS | -ve | Safe | 2000-6000 | Slight T to safe | Safe | T | T | T | T | NT | T | NC | C | NC | NC |
| Norgestrel | NT | NT | NT | NT | NS | -ve | Safe | 5010-11200 | Safe | Safe | T | T | T | T | NT | T | NC | C | NC | NC |
| Nystatin | NT | NT | NT | T | NS | -ve | T | 10000 | Safe | Safe | T | NT | T | T | NT | T | C | NC | NC | NC |
| Ofloxacin | NT | T | NT | T | NS | -ve | T | 3590-5450 | Slight T to safe | Safe | NT | T | T | T | NT | T | C | NC | NC | NC |
| Omeprazole | NT | NT | NT | T | NS | -ve | T | 2210-4000 | Slight T | T | T | NT | NT | NT | NT | T | C | C | C | C |
| Orphenadrine | T | T | T | NT | S | +ve | T | 100-255 | Moderate T | T | T | NT | NT | NT | NT | T | C | C | C | C |
| Oxymetazoline | T | T | NT | T | S | -ve | T | 0.68-48 | Highest T | T | NT | NT | T | T | NT | T | C | C | C | C |
| Paclitaxel | NT | NT | NT | T | NS | -ve | T | 32530 | Safe | Safe | T | NT | T | NT | NT | T | C | NC | NC | NC |
| Paracetamol | NT | T | NT | NT | NS | -ve | T | 338-1944 | Moderate to slight T | T | T | T | T | T | NT | T | C | C | C | C |
| Paraldehyde | NT | T | NT | T | NS | +ve | T | 1300-5000 | Slight T to safe | Safe | T | NT | T | NT | NT | T | C | NC | NC | NC |
| Pethidine | T | T | T | NT | NS | -ve | T | 162 | Moderate T | T | NT | NT | T | NT | NT | T | C | C | C | C |
| Phenobarbital | NT | T | NT | T | NS | -ve | T | 162 | Moderate T | T | T | NT | NT | NT | NT | T | C | C | C | C |
| Phenothiazine | NT | T | T | NT | NS | +ve | T | 1370-2000 | Slight T | T | T | T | T | T | NT | T | C | C | C | C |
| Phenoxymethylpenicillin | NT | T | NT | T | S | -ve | T | 1040-2220 | Slight T | T | T | NT | NT | T | NT | T | C | C | C | C |
| Phenylephrine | NT | T | T | NT | S | -ve | T | 17-350 | High to moderate T | T | NT | NT | T | NT | NT | T | C | C | C | C |
| Phenytoin | NT | NT | NT | T | NS | -ve | T | 1635 | Slight T | T | T | NT | T | NT | NT | T | C | C | C | C |
| Pilocarpine | T | T | NT | T | S | -ve | T | 402 | Moderate T | T | NT | NT | T | NT | NT | T | C | C | C | C |
| Piperacillin | NT | NT | NT | T | NS | -ve | T | 2260-10000 | Slight T to Safe | Safe | T | NT | T | T | NT | T | C | NC | NC | NC |
| Podophyllin resin | NT | T | T | NT | NS | -ve | T | 68 | Highest to moderatr T | T | T | NT | NT | T | NT | T | C | C | C | C |
| Postinor-2/Levonorgestrel | NT | NT | NT | NT | NS | -ve | Safe | 5000 | Safe | Safe | T | T | T | T | NT | T | NC | C | NC | NC |
| Potassium Chloride | NT | T | T | T | NS | +ve | T | 117-3020 | Moderate to slight T | T | NT | NT | NT | NT | NT | Safe | NC | C | NC | NC |
| Potassium permanganate | NT | T | T | T | S | +ve | T | 1090-2000 | Slight T | T | NT | NT | T | NT | NT | T | C | C | C | C |
| Pralidoxime mesylate | T | T | NT | T | S | -ve | T | 90-4100 | Moderate to Slight T | T | T | T | T | NT | NT | T | C | C | C | C |
| Praziquantel | NT | T | NT | T | NS | -ve | T | 2840 | Slight T | T | T | NT | NT | NT | NT | T | C | C | C | C |
| Prazosin | NT | T | NT | T | NS | -ve | T | 102-1950 | Moderate to slight T | T | T | T | NT | T | NT | T | C | C | C | C |
| Prednisolone | NT | NT | NT | NT | NS | -ve | Safe | 65-3500 | Moderate to slight T | T | T | T | T | T | NT | T | NC | NC | C | NC |
| Procarbazine | NT | T | NT | NT | NS | -ve | T | 785 | Slight T | T | T | T | T | T | NT | T | C | C | C | C |
| Prochlorperazine | T | T | T | NT | S | +ve | T | 191-750 | Moderate T | T | T | NT | T | NT | NT | T | C | C | C | C |
| Procyclidine | T | T | NT | NT | NS | -ve | T | 60 | Moderate T | T | T | NT | T | NT | NT | T | C | C | C | C |
| Progesterone | NT | NT | NT | NT | NS | -ve | Safe | 327 | Moderate T | T | T | T | T | T | NT | T | NC | NC | C | NC |
| Proguanil | T | T | T | T | S | -ve | T | 58 | Moderate T | T | T | T | T | NT | NT | T | C | C | C | C |
| Prohance/Gadoteridol | NT | NT | NT | T | NS | -ve | T | 6145-7822 | Safe | Safe | NT | NT | T | T | NT | T | C | NC | NC | NC |
| Promethazine | T | T | NT | T | S | -ve | T | 160-400 | Moderate T | T | NT | T | T | T | NT | T | C | C | C | C |
| Propranolol HCL | NT | T | NT | NT | S | -ve | T | 660 | Slight T | T | NT | T | NT | NT | NT | T | C | C | C | C |
| Pyrazinamide | NT | NT | NT | NT | NS | -ve | Safe | 1680-2973 | Slight T | T | NT | NT | NT | NT | NT | Safe | C | NC | NC | NC |
| Pyridoxine | NT | NT | NT | NT | S | -ve | T | 4000 | Slight T | T | NT | NT | NT | NT | NT | Safe | NC | C | NC | NC |
| Pyrimethamine | T | T | NT | T | S | +ve | T | 440 | Moderate T | T | T | T | NT | T | NT | T | C | C | C | C |
| Quinine | NT | T | NT | NT | S | -ve | T | 115 | Moderate T | T | NT | NT | NT | NT | NT | Safe | NC | C | NC | NC |
| Ranitidine | NT | NT | NT | T | S | -ve | T | 77-5000 | Moderate to safe | Safe | T | NT | T | T | NT | T | C | NC | NC | NC |
| Reserpine | NT | T | NT | T | NS | -ve | T | 7-420 | Highest to moderate T | T | T | NT | T | T | NT | T | C | C | C | C |
| Rifampicin | NT | NT | NT | T | NS | -ve | T | 1570 | Slight T | T | T | T | T | T | NT | T | C | C | C | C |
| Risperidone | NT | T | NT | T | NS | -ve | T | 34-57.7 | Highest T | T | NT | T | NT | NT | NT | T | C | C | C | C |
| Ropivacaine | T | NT | NT | NT | NS | -ve | T | 9.9-980 | Slight T | T | T | NT | T | T | NT | T | C | C | C | C |
| Salbutamol | NT | T | NT | NT | S | -ve | T | 1100 | Slight T | T | NT | NT | T | T | NT | T | C | C | C | C |
| Silver nitrate | T | T | T | T | S | +ve | T | 2000-3702 | Slight T | T | NT | T | T | NT | NT | T | C | C | C | C |
| Silver sulphadiazine | NT | NT | NT | T | S | -ve | T | 10001 | safe | Safe | T | T | T | T | NT | T | C | NC | NC | NC |
| Simvastatin | NT | NT | NT | NT | NS | -ve | Safe | 4438 | Slight T | T | NT | NT | T | NT | NT | T | NC | NC | C | NC |
| Sodium amidotrizoate/Diatrizoate | NT | NT | NT | T | NS | -ve | T | 7000 | Safe | Safe | T | T | T | T | NT | T | C | NC | NC | NC |
| Sodium ascorbate | NT | NT | NT | NT | NS | -ve | Safe | 5000-16500 | Safe | Safe | T | T | T | T | NT | T | NC | C | NC | NC |
| Sodium Bicarbonate | NT | T | NT | T | NS | +ve | T | 3400-7570 | Slight T to safe | Safe | NT | NT | NT | NT | NT | Safe | NC | NC | C | NC |
| Sodium chloride | NT | T | T | T | NS | +ve | T | 3000-10000 | Slight T to safe | Safe | NT | NT | NT | NT | NT | Safe | NC | NC | C | NC |
| Sodium cromoglycate/Cromoglicic acid | NT | NT | T | NT | NS | -ve | T | 11000 | Safe | Safe | NT | T | NT | T | NT | T | C | NC | NC | NC |
| Sodium lactate/Lactic acid | NT | NT | NT | T | NS | +ve | T | 2000-4936 | Slight T | T | NT | NT | NT | NT | NT | Safe | NC | C | NC | NC |
| Sodium thiosulphate/Thiosulphuric acid | NT | NT | NT | NT | NS | +ve | T | 2000-5000 | Slight T to safe | Safe | NT | NT | NT | NT | NT | Safe | NC | NC | C | NC |
| Sodium Valproate | NT | NT | NT | NT | NS | +ve | T | 670-1098 | Slight T | T | NT | NT | NT | NT | NT | Safe | NC | C | NC | NC |
| Spironolactone | NT | NT | NT | NT | NS | -ve | Safe | 1000 | Slight T | T | T | T | T | T | NT | T | NC | NC | C | NC |
| Stavudine | NT | NT | NT | T | NS | -ve | T | 4000 | Slight T | T | NT | NT | NT | NT | NT | Safe | NC | C | NC | NC |
| Streptomycin | NT | NT | NT | T | NS | -ve | T | 430-9000 | Moderate T to safe | Safe | T | T | T | NT | NT | T | C | NC | NC | NC |
| Sulfamethoxazole | NT | NT | NT | T | NS | -ve | T | 2300-6200 | Slight T to safe | Safe | T | T | T | T | NT | T | C | NC | NC | NC |
| Sulphadoxine | NT | NT | NT | T | NS | -ve | T | 5200 | Safe | Safe | T | T | T | T | NT | T | C | NC | NC | NC |
| Sulphur/Octasulfur | T | T | T | T | NS | +ve | T | 2000-12448 | Slight T to safe | Safe | T | NT | T | NT | NT | T | C | NC | NC | NC |
| Suramin | NT | NT | NT | T | NS | -ve | T | 750 | Slight T | T | T | T | T | T | NT | T | C | C | C | C |
| Suxamethonium | T | NT | T | NT | NS | -ve | T | 0.24-125 | Highest to moderate T | T | T | NT | T | NT | NT | T | C | C | C | C |
| Tamoxifen | T | T | T | NT | NS | -ve | T | 4100 | Slight T | T | T | NT | NT | NT | NT | T | C | C | C | C |
| Taxotere/Docetaxel | NT | NT | NT | T | NS | -ve | T | 2000 | Slight T | T | T | T | T | NT | NT | T | C | C | C | C |
| Tazobactam | NT | NT | NT | T | NS | -ve | T | 5000 | Safe | Safe | T | NT | NT | T | NT | T | C | NC | NC | NC |
| Tenofovir | NT | NT | NT | T | NS | -ve | T | 30-1500 | Highest to slight T | T | T | T | T | NT | NT | T | C | C | C | C |
| Tetracycline | NT | T | NT | T | NS | -ve | T | 808 | Slight T | T | T | T | T | T | NT | T | C | C | C | C |
| Theophylline | NT | T | NT | T | NS | -ve | T | 225-2000 | Moderate to slight T | T | NT | NT | NT | NT | NT | Safe | NC | C | NC | NC |
| Thiamine | NT | NT | NT | T | NS | -ve | T | 3710-8224 | Slight T to safe | Safe | T | T | T | NT | NT | T | C | NC | NC | NC |
| Thyroxine sodium | NT | NT | NT | NT | NS | -ve | Safe | 10000 | Safe | Safe | NT | NT | T | T | NT | T | NC | C | NC | NC |
| Timolol | NT | T | NT | T | NS | -ve | T | 1028-1137 | Slight T | T | NT | T | NT | NT | NT | Safe | NC | C | NC | NC |
| Tramadol | NT | T | NT | NT | NS | -ve | T | 228-350 | Moderate T | T | T | NT | NT | NT | NT | T | C | C | C | C |
| Tranexamic acid | NT | NT | NT | NT | NS | -ve | Safe | 10000 | safe | Safe | T | NT | NT | T | NT | T | NC | C | NC | NC |
| Travatan (Travoprost) | NT | T | NT | NT | S | -ve | T | 65 | Highest to moderate T | T | NT | NT | T | NT | NT | T | C | C | C | C |
| Triamcinolone acetonide | NT | NT | NT | NT | NS | -ve | Safe | 13.1-2168 | Highest to slight T | T | T | T | T | T | NT | T | NC | NC | C | NC |
| Trimethoprim | NT | NT | NT | NT | NS | -ve | Safe | 2764-5300 | Slight T to safe | Safe | T | T | NT | NT | NT | T | NC | C | NC | NC |
| Tropicamide | T | T | NT | NT | NS | -ve | T | 865 | Slight T | T | T | NT | T | NT | NT | T | C | C | C | C |
| Vancomycin | NT | NT | NT | T | NS | -ve | T | 319-5000 | Moderate to safe | Safe | T | T | T | T | NT | T | C | NC | NC | NC |
| Vecuronium bromide | NT | T | NT | T | NS | -ve | T | 455 | Moderate T | T | T | NT | T | NT | NT | T | C | C | C | C |
| Verapamil | NT | T | NT | NT | NS | -ve | T | 150-163 | Moderate T | T | T | NT | NT | NT | NT | T | C | C | C | C |
| Vincristine | NT | T | NT | T | NS | -ve | T | 5.2-1300 | Highest to slight T | T | T | T | T | T | NT | T | C | C | C | C |
| Vitamin A | NT | NT | NT | NT | NS | +ve | T | 1100-26000 | Slight T to safe | Safe | T | T | T | T | NT | T | C | NC | NC | NC |
| Vitamin B12 | NT | NT | NT | T | NS | -ve | T | 5000 | Safe | Safe | NT | NT | T | NT | NT | T | C | NC | NC | NC |
| Vitamin K | NT | NT | NT | NT | S | -ve | T | 41,000-52000) | safe | Safe | T | T | T | T | NT | T | C | NC | NC | NC |
| Warfarin sodium | NT | T | T | NT | NS | -ve | T | 1-1400 | Highest to slight T | T | T | T | T | NT | NT | T | C | C | C | C |
| Xalatan (Latanoprost) | NT | NT | NT | NT | S | -ve | T | 0.05-50 | Highest T | T | NT | NT | T | NT | NT | T | C | C | C | C |
| Zidovudine | NT | NT | NT | T | NS | -ve | T | 3084 | Slight T | T | NT | T | T | NT | NT | T | C | C | C | C |
| Zinc oxide | T | T | T | T | NS | +ve | T | 2000-7950 | Slight T to safe | Safe |  |  |  |  |  |  |  | NC |  | NC |
|  | 54 | 143 | 58 | 163 | 76 | 39 | 255 |  |  | 205 | 201 | 146 | 211 | 145 | 2 | 264 | 235 | 205 | 188 | 169 |
|  | 296 | 296 | 296 | 296 | 296 | 296 | 296 |  |  | 293 | 294 | 294 | 294 | 294 | 294 | 294 | 294 | 293 | 292 | 296 |

Key: T = Toxic, NT = Non-toxic, -ve = negative = Non-toxic, +ve = positive = Toxic, S = Sensitizer = Toxic, NS = Non-sensitizer = Non-Toxic.

C = Consensus, NC = Non-consensus.
